# A Droplet Microfluidic System to Fabricate Hybrid Capsules Enabling Stem Cell Organoid Engineering

**DOI:** 10.1002/advs.201903739

**Published:** 2020-04-11

**Authors:** Haitao Liu, Yaqing Wang, Hui Wang, Mengqian Zhao, Tingting Tao, Xu Zhang, Jianhua Qin

**Affiliations:** ^1^ H. Liu, Y. Wang, H. Wang, M. Zhao, T. Tao, Prof. J. Qin Division of Biotechnology CAS Key Laboratory of SSAC Dalian Institute of Chemical Physics Chinese Academy of Sciences Dalian 116023 China; ^2^ H. Liu, Y. Wang, H. Wang, M. Zhao, T. Tao, Prof. J. Qin University of Chinese Academy of Sciences Beijing 100049 China; ^3^ Dr. X. Zhang Department of Biomedical Engineering Duke University Durham NC 27708 USA; ^4^ Prof. J. Qin Institute for Stem Cell and Regeneration Chinese Academy of Sciences Beijing 100101 China; ^5^ Prof. J. Qin CAS Center for Excellence in Brain Science and Intelligence Technology Chinese Academy of Sciences Shanghai 200031 China

**Keywords:** all‐in‐water systems, droplet microfluidics, hydrogel capsules, islet organoids, stem cells

## Abstract

Organoids derived from self‐organizing stem cells represent a major technological breakthrough with the potential to revolutionize biomedical research. However, building high‐fidelity organoids in a reproducible and high‐throughput manner remains challenging. Here, a droplet microfluidic system is developed for controllable fabrication of hybrid hydrogel capsules, which allows for massive 3D culture and formation of functional and uniform islet organoids derived from human‐induced pluripotent stem cells (hiPSCs). In this all‐in‐water microfluidic system, an array of droplets is utilized as templates for one‐step fabrication of binary capsules relying on interfacial complexation of oppositely charged Na‐alginate (NaA) and chitosan (CS). The produced hybrid capsules exhibit high uniformity, and are biocompatible, stable, and permeable. The established system enables capsule production, 3D culture, and self‐organizing formation of human islet organoids in a continuous process by encapsulating pancreatic endocrine cells from hiPSCs. The generated islet organoids contain islet‐specific α‐ and β‐like cells with high expression of pancreatic hormone specific genes and proteins. Moreover, they exhibit sensitive glucose‐stimulated insulin secretion function, demonstrating the capability of these binary capsules to engineer human organoids from hiPSCs. The proposed system is scalable, easy‐to‐operate, and stable, which can offer a robust platform for advancing human organoids research and translational applications.

## Graphical abstract

Human organoids derived from self‐organizing stem cells represent a major technological breakthrough with the potential to revolutionize biomedical research. Here, a strategy is presented for fabricating hydrogel capsules in a droplet microfluidic system that enables 3D culture, growth, and massive generation of uniform islet organoids from human‐induced pluripotent stem cells in a reproducible manner to facilitate organoids research and therapy.

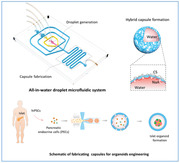

There has been a growing interest in building functional 3D organ models in vitro. Organoids are multicellular tissues formed by self‐organization of stem cells in 3D culture, which can mimic corresponding in vivo organ. They represent a major technological breakthrough in creating a new class of organ models.^[^ ^1^, ^2^, ^3^ ^]^ Recent significant progresses have enabled successful generation of various organoids, such as intestine,^[^ ^4^, ^5^, ^6^ ^]^ brain,^[^ ^7^, ^8^, ^9^ ^]^ liver,^[^ ^10^, ^11^, ^12^ ^]^ kidney,^[^ ^13^, ^14^, ^15^ ^]^ and retina.^[^ ^16^, ^17^, ^18^ ^]^ They have shown enormous potential in organ developmental studies, disease modeling, drug screening, and translational research.^[^ ^15^, ^19^, ^20^, ^21^ ^]^ Generally, organoid derivation protocols mainly rely on self‐organization of stem cells in 3D animal‐derived matrices such as Matrigel in a multi‐step process. However, organoids are often limited by significant variability and low throughput, which is partially due to the complex and poorly defined matrix, as well as the labor‐intensive multistep process in the existing approaches.

Advances in biomaterials and microfabrication technologies have offered great opportunities to recreate 3D tissue/organ models with more physiological relevance in a controllable manner.^[^ ^22^ ^]^ Currently, several groups have attempted to utilize hydrogels as biomimetic matrices^[^ ^4^, ^15^, ^23^, ^24^, ^25^ ^]^ or scaffolds^[^ ^12^, ^23^, ^26^ ^]^ for organoid formation to reduce their variability. These hydrogels have been fabricated into different modules with flexible format, such as porous scaffolds,^[^ ^12^ ^]^ patterned substrates,^[^ ^23^ ^]^ and microfibers,^[^ ^26^ ^]^ to support 3D organoids culture. Despite the progress, achieving high‐fidelity and reproducible organoid models remains challenging.

Hydrogel capsules have been recognized as proper 3D culture scaffolds and transplanted cell‐laden carriers in tissue engineering owing to their uniform morphology, proper permeability, and the ability in scale‐up production.^[^ ^27^, ^28^, ^29^ ^]^ Herein, we make the first attempt to develop a novel droplet microfluidic system for one‐step fabrication of hybrid hydrogel capsules that allow for 3D culture, growth, and generation of hiPSCs‐derived organoids in a reproducible and high‐throughput manner. The droplet microfluidic system contains different functional units, including multi‐phase fluid inlets, droplet generation, and capsule fabrication units (**Figure** **1**A). The multi‐phase fluid inlets are utilized to pump core flow (dextran, DEX with NaA), middle flow (polyethylene glycol, PEG), and shell flow (PEG with CS) into the corresponding microchannels. The droplet generation and capsule fabrication units are designed to form droplet templates and hybrid capsules, respectively. Generally, hydrogel capsules can be fabricated in microfluidic systems with the existence of organic solvent that might cause protein or enzyme denaturation, inhibit cell growth, and block mass transfer in subsequent cell culture.^[^ ^30^, ^31^, ^32^ ^]^ Thus, multiple post‐processing washing steps are inevitable in such a system, which is time/labour‐consuming and lead to potential contamination or negative effects on cell viability.^[^ ^33^, ^34^ ^]^ To improve the biocompatibility of the droplet system and reduce the post‐processing steps, hydrogel capsules are fabricated under a mild all‐in‐water condition in our system.

**Figure 1:**
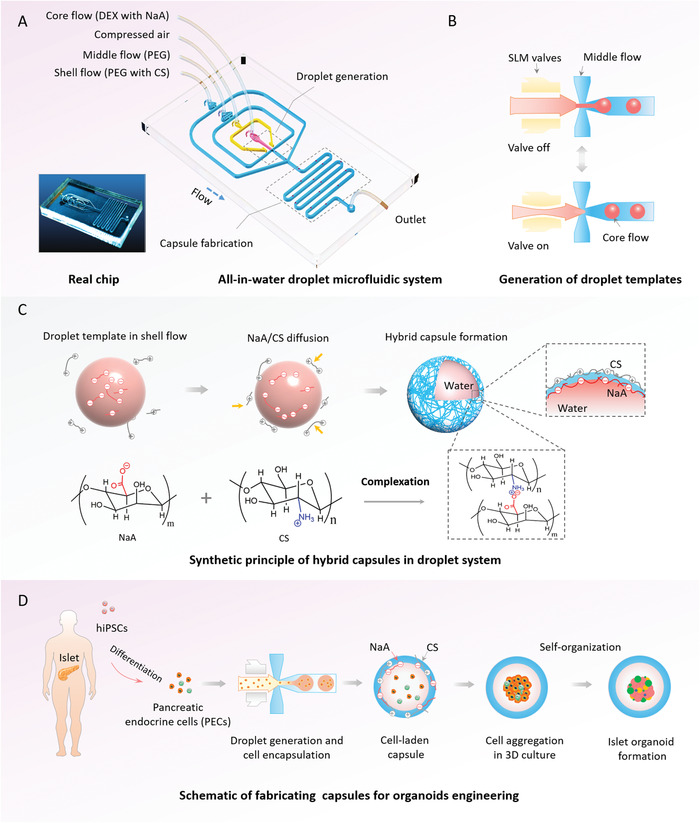
Schematic diagram of all‐in‐water droplet microfluidic system to fabricate hybrid hydrogel capsules that enable 3D culture and generation of islet organoids. A) Real chip and configuration of the droplet microfluidic chip, including shell flow, middle flow, core flow, compressed air, droplet generation unit, reaction unit, and outlet. B) The procedures for generation of droplet templates with the auxiliary of single layer membrane (SLM) in the droplet generation unit. C) A diagram of hybrid capsules synthetic principle based on the complexation of Na‐alginate (NaA) and chitosan (CS) in reaction unit. D) The process of the capsules utilized for human‐induced pluripotent stem cell (hiPSC)‐derived islet organoids engineering.

To facilitate controllable generation of droplet templates, pneumatic single‐layer membrane (SLM) valves are integrated into this system (Figure 1B and Movie S1, Supporting Information), which could partially address the inherent limitation of low interfacial tension^[^ ^35^ ^]^ in all‐in‐water systems. In this system, the capsules are synthesized using diffusion and interfacial complexation of oppositely charged NaA and CS in a hybrid fashion (Figure 1C), thereby improving the stability of the capsules for 3D cell culture. Specifically, alginate‐based capsules can be formed with divalent cations (e.g., calcium) in a reversible manner, leading to hydrogel disintegration or swelling in physiological or cell culture conditions due to the exchange reactions between Ca^2+^ and the surrounding monovalent cations, such as Na^2+^.^[^ ^36^ ^]^ However, the chitosan‐coated alginate shows higher stability in salt solutions (e.g., phosphate buffer solutions, PBS),^[^ ^37^ ^]^ which can partially address the aforementioned drawbacks of alginate hydrogels. In addition, hundreds to thousands of capsules can be produced within 1 min (Figure S1, Supporting Information) in the proposed system, indicating the potential of this system in fabricating 3D scaffolds in a high‐throughput manner.

To maintain the uniform size and morphology of produced capsules in a controllable fashion, we further explored the effects of key parameters in the all‐in‐water droplet system on the characteristics of the binary capsules. Generally, flow rate is one of the major factors that might affect the size of droplets generated in the microfluidic system. Initially, different core flow rates ranging from 0.1 to 0.4 µL min^−1^ were examined. As shown in **Figure** **2**A,B, the diameter of capsules increases significantly with the increase of core flow rate, while no significant changes are observed in the size of capsules with the change of middle and shell flow rates (Figure S2, Supporting Information). Furthermore, the fabricated capsules exhibit monodispersed features with a relatively small coefficient of variation (CV < 5%), indicating the potential of the system to produce uniform scaffolds (Figure 2B and Figure S2B,D, Supporting Information). Since the droplet templates were generated using the SLM valves, we also examined the effects of valve switch frequency on the size of capsules. Here, the valve switch frequency is defined as the total time of valve being switched on and off. As shown in Figure 2C,D, capsules with larger diameter are produced with only a minimal change in valve frequency due to mass conservation,^[^ ^38^ ^]^ demonstrating the significant effects of valve frequency on capsule size. As such, customized capsules with specific sizes can be prepared by simply adjusting core flow rate and valve frequency in the system.

**Figure 2:**
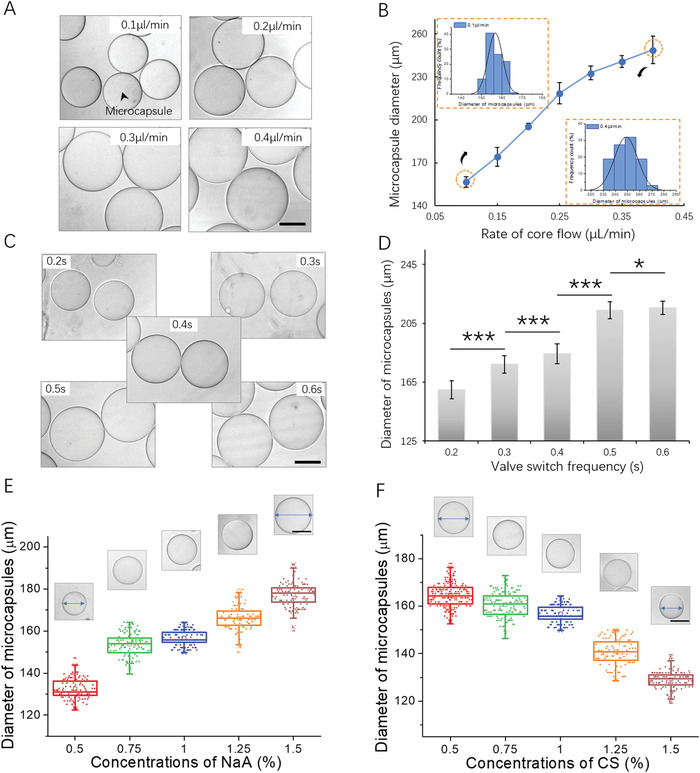
Effects of different fluid parameters on the diameter and polydispersity of capsules. A) The images of capsules generated under serial rates of core flow, ranging from 0.1 to 0.4 µL min^−1^. B) The capsule diameter as a function of the rates of core flow and the size distribution of capsules generated under boundary flow rates (0.1 and 0.4 µL min^−1^). C) Bright‐field images of capsules fabricated under serial valve switch frequencies, ranging from 0.2 to 0.6 s. D) Diameter profiles of capsules obtained using different valve switch frequencies. Data are shown as mean ± SD. Student's *t*‐tests are performed. *p < 0.05, ***p < 0.001. The images and plot of capsules generated using different concentrations of E) NaA and F) CS, respectively. Quantitative analysis of the diameter of capsules are performed on at least 50 capsules. Scale bars: 100 µm.

In addition, the diffusion of NaA and CS on the droplet template surface is crucial for the formation of capsules within this all‐aqueous system. The driving force of diffusion is the concentration gradient according to Fick's first law ($\text{J}\;\text{=}\;-\text{D}\;\left(\frac{\partial \text{C}}{\partial \text{X}}\right)$,with J being diffusion flux, *D* being diffusion coefficient, and $\frac{\partial \text{C}}{\partial \text{X}}$ being concentration gradient). We hypothesized that the variation of NaA/CS concentrations could affect capsule size, which was verified by preparing 0.5% w/w to 1.5% w/w NaA/CS solutions to fabricate hybrid capsules. As shown in Figure 2E, capsules with lager diameter were produced as the concentration of NaA increased. On the contrary, the diameter of capsules decreased significantly with the increasing CS concentration (Figure 2F). Raising NaA concentration leads to an increase of diffusion flux. Therefore, the complexation of NaA and CS occurs far away from the droplet template surface, resulting in capsules with a larger size. Conversely, a higher diffusion flux of CS results in complexation closer to the droplet surface, leading to the production of capsules with a smaller size.

The hollow cores of capsules have been known to be beneficial for encapsulating cells and shaping them into uniform spheroids. To evaluate the core–shell structure of hybrid capsules, the fluorescein isothiocyanate (FITC)‐labeled and freeze‐dried capsules are investigated in the following experiments. FITC‐labeled capsules are synthesized with FITC‐chitosan that is located on the edge of capsules (**Figure** **3**A–C), indicating the well‐defined compositions of the capsules. Furthermore, sliced FITC‐labeled and freeze‐dried capsules exhibit hollow cores (Figure 3D–H), demonstrating the core–shell structure of binary capsules. Particularly, the thin shell is presented in the SEM images, of which the thickness is below 1 µm (Figure 3D–F). The thickness of shell is nearly independent of the multi‐flow rates and concentration of NaA/CS in this system due to the limited diffusion of NaA/CS after capsule fabrication. This might result in limited controllability on the capsule architectures in the fabrication process, but the thickness of capsule shell can be adjusted by postprocessing, such as layer‐by‐layer deposition of the oppositely charged polymers according to the previous reports.^[^ ^39^ ^]^ As such, the distance between different cellular spheroids within capsules packed in the same Petri dish can be precisely controlled, thus affecting cell–cell interactions within different capsules. In addition, capsules with proper permeability allow for exchange of nutrients, gases, and metabolites between cells and the external microenvironment, as well as protection of the cells from mechanical damage and immune rejection. The permeability of capsules is characterized by using fluorescein sodium (376 Da), albumin from bovine serum (BSA)‐FITC (67 kDa), and dextran‐FITC (500 kDa) as representative indicators of small molecules, proteins, and macromolecule, respectively. The newly fabricated capsules were immersed in solutions of these fluorescent molecules that diffused into hybrid capsules. The diffusion of fluorescein sodium reaches its equilibrium state (permeability value: 0.02 µm s^−1^) within 20 min, indicating the high permeability of capsules to small molecules (Figure 3I and Figure S3, Supporting Information). Similarly, BSA‐FITC can partially diffuse into capsules and reaches an equilibrium state, with a small permeability value of 2.1 × 10^−3^ µm s^−1^. On the contrary, few dextran‐FITC molecules can enter the capsules even after an overnight treatment.

**Figure 3:**
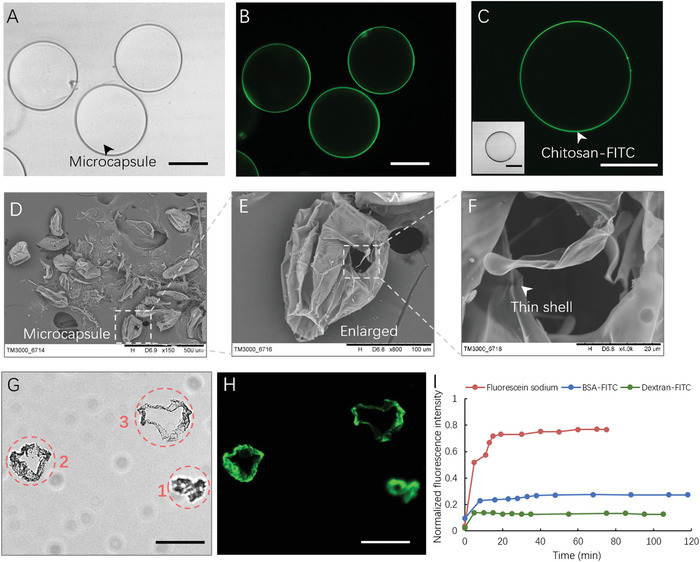
Characterization of the structure and permeability of hybrid capsules. A) Bright‐field image of the hydrogel capsules. B,C) Fluorescence image of hydrogel capsules. D–F) SEM images of freeze‐dried capsules. G) Bright‐field image of frozen section capsules. Images 1–3 represent different cross sections of distorted capsules. H) Fluorescence image of frozen section capsules. I) Fluorescence curves of fluorescein sodium (red), BSA‐FITC (blue), and dextran‐FITC (green) diffuse in hydrous capsules. Data is normalized with the fluorescence intensity of the area outside the capsules. Scale bars: 100 µm. All the capsules are fabricated under the same conditions (rates of core, middle, and shell flow: 0.15, 2, and 4 µL min^−1^; valve frequency: 0.4 s; concentrations of NaA/CS: 1% w/w).

As above, the fabricated capsules in droplet microfluidic system display the proper permeability and uniformity in a controllable way. In order to assess the capability of such capsules for cell encapsulation and 3D culture, cell lines (e.g., mouse islet β‐TC6) are initially resuspended in the core flow and infused in the hybrid capsules. Herein, these capsules could maintain the integrity for at least 25 days under cell culture conditions, demonstrating the stability of hydrogel capsules for 3D cell culture (Figure S4, Supporting Information). As shown in Figure S5A,B, Supporting Information, the encapsulated β‐TC6 cells aggregate into smooth spheroids, which keep growing during 10 days of culture until fill in the capsules. This data indicates that the hybrid capsules with controlled size can constrain the shape and overgrowth of spheroid structures. Such cellular spheroids are generated in a high‐throughput manner, which enables hundreds to thousands of capsules to be produced within 1 min (Figures S1 and S5A, Supporting Information). Compared with the cellular spheroids produced with other methodology, such as micro‐wells^[^ ^40^ ^]^ and hanging drop,^[^ ^41^ ^]^ these spheroids hold more promise in their reproducible generation, higher uniformity, and potential cell therapy applications due to the inherent advantages of droplet microfluidic and the presence of capsules. Moreover, similar to previous reports,^[^ ^40^, ^41^ ^]^ the spheroids exhibit favorable cell viability and proliferation ability during the culture period (Figure S5C, Supporting Information), confirming the biocompatibility and the feasibility of 3D cell culture of these hydrogel scaffolds. In addition, islet‐specific function is identified by immunostaining for hormonal protein marker in β‐TC6 spheroids within capsules, which are found to express insulin (Figure S5D, Supporting Information). These results indicate that the hybrid capsules are beneficial for cell encapsulation and aggregation to form cellular spheroids, as well as allow for the proliferation of β‐TC6 cells.

Human‐induced pluripotent stem cells (hiPSCs) are rooted in somatic cells (e.g., fibroblasts) by cell reprogramming, which have the capability of generating various organoids such as intestine, brain, liver, and islet via self‐renewal and self‐organization of stem cells in 3D culture.^[^ ^42^, ^43^ ^]^ Pancreatic islet is an important secretory gland with exocrine and endocrine function and holds the important role of blood glucose homeostasis in vivo. Islet organoids derived from stem cells hold great potential in diabetes research, drug testing, and cell therapy. In the following experiment, we investigated the feasibility of binary capsules as 3D scaffolds for engineering islet organoids from hiPSCs in a controlled format. Prior to the formation of organoids, human hiPSCs are sequentially induced and differentiated into endoderm, pancreatic progenitors, and endocrine cells by addition of different chemical factors on Petri dish according to our previous report.^[^ ^44^ ^]^ Then, the differentiated pancreatic endocrine cells are resuspended in NaA of core flow and encapsulated into hybrid capsules (Figures 1D and 4A). It is known that the intercellular junctions of pancreatic endocrine cells are crucial in regulating islet cell aggregation and organization.^[^ ^45^, ^46^ ^]^ The endocrine cells are found to aggregate and form single complete spheroids with smooth edge after encapsulation in hybrid capsules by day 1 (**Figure** **4**B). At present, most of the existing methods for organoid generation often rely on self‐organization of cells in low‐adhesion Petri dishes, which might lead to organoid formation with higher variation in size. However, the cellular spheroids generated in capsules can self‐organize into islet organoids with more uniform size and a relatively narrow particle size distribution (CV <9%) than that of islet organoids cultured in Petri dishes (Figure S6, Supporting Information), demonstrating the efficient generation of reproducible organoids in hybrid capsules using the droplet microfluidic system. In addition, the size of islet organoids varied significantly as the encapsulated cell density changes (Figure S7, Supporting Information), indicating the capability of hybrid capsules to constrain the shape and size of the organoids.

**Figure 4:**
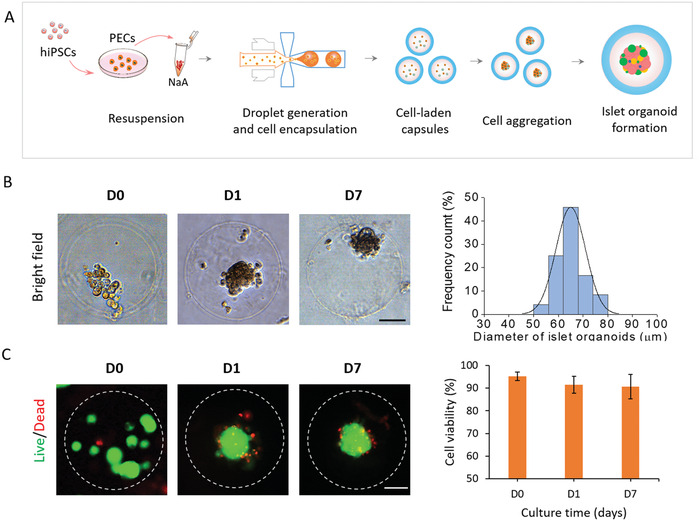
The encapsulation and formation of hiPSC‐derived islet organoids in the hybrid capsules. A) Flow chart of hybrid capsules used for the encapsulation of differentiated pancreatic endocrine cells from hiPSCs and the formation of islet organoids. B) Representative images of islet cell spheroids after encapsulation in capsules for 0, 1, and 7 days. Islet organoids are formed by self‐organization and further differentiation of pancreatic endocrine cells. Size distribution is assessed by the diameter of organoids on day 1. C) Cell viability of islet organoids is evaluated at days 0, 1, and 7 of culture in capsules by Live/Dead staining and fluorescence quantitative analysis. The green and red fluorescence represent live and dead cells, respectively. Scale bars: 50 µm.

To quantitatively evaluate the growth of human islet organoids in capsules, we measured the size of organoids after 1 day and 7 days of culture in 3D conditions. Most islet organoids presented an average diameter of 60–70 µm during 3D culture in hybrid capsules. These data indicate organoids can maintain controllable size, which may improve the reproducibility of organoids. In addition, the cell viability of organoids is examined and measured to be more than 90% on days 0, 1, and 7, demonstrating the favorable viability of organoids in capsules (Figure 4C). These data indicate that the mild all‐aqueous liquid environment is highly biocompatible for cell survival, assembly, and organoid formation in capsules. In addition, we calculated the percentages of capsules containing living organoids at day 0 (92%) and day 7 (89%) to further evaluate the high efficiency of encapsulation progress and stability of the capsules for organoid culture (Figure S8, Supporting Information). Moreover, the proper permeability of capsules to small molecules and proteins are in favor of nutrient exchange and soluble factors' delivery, thereby supporting the efficient growth and culture of organoids.

In vivo, pancreatic islets are multicellular tissues mainly consisting of islet‐specific β‐ and α‐cells, which can secrete several pancreas‐specific hormones, including insulin (INS), glucagon (GCG), and pancreatic polypeptide (PPY) to maintain blood glucose homeostasis. To identify the differentiated characterization of hiPSC‐derived islet organoids generated in hybrid capsules, the gene expression profiles of pancreatic lineage and endocrine cell maturation are examined by real‐time PCR (**Figure** **5**B). Notably, the islet β‐cell associated transcriptional factor (NKX6.1), insulin secretion function associated marker (INS), α‐cell specific marker (GCG and PPY) display remarkably higher expression in organoids than in undifferentiated hiPSCs, indicating the presence of multiple endocrine lineages in islet organoid. In addition, the encapsulated organoids showed significantly higher expression of these related markers than that in 2D cells cultured in Petri dishes, demonstrating the capability of hydrogel capsules for engineering functional islet organoids. Similarly, hormone‐associated markers (INS, NKX6.1, GCG, PPY) are examined in organoids using immunostaining assay (Figure 5A). These organoids exhibit obvious expression of hormone proteins that is negative in undifferentiated hiPSCs (Figure S9, Supporting Information), further demonstrating the cellular heterogeneity and hormone secretion of human islet organoids that are analogous to human pancreatic islet in vivo. In addition, the expressions of ISN, NKX6.1 and GCG were quantitatively analyzed for the percentage of INS^+^, NKX6.1^+^ and GCG^+^ cells in islet organoids within capsules (Figure 5C).

**Figure 5:**
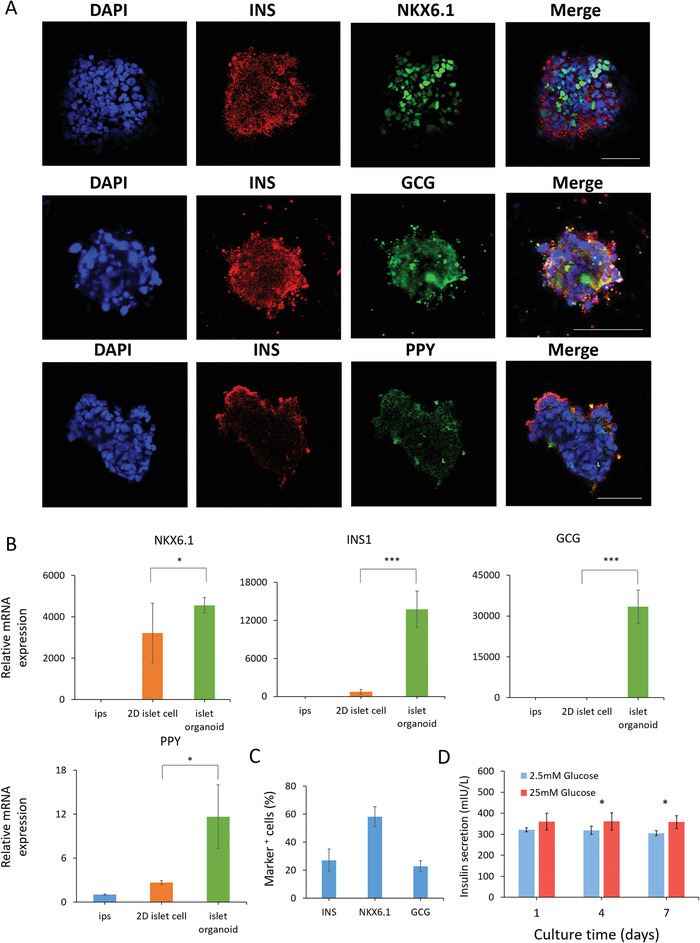
Identification of differentiation and functions of islet organoids cultured in hybrid capsules. A) Immunohistochemical staining of pancreatic endocrine hormone markers INS, NKX6.1, GCG, and PPY in islet organoids after 7 days of encapsulation in capsules. DAPI stains the nuclei (blue). Scale bars: 50 µm. B) The expressions of β cell‐associated transcriptional factor marker (NKX6.1) and pancreatic endocrine hormone genes (INS, GCG, PPY) are examined in hiPSCs, islet cells in petri dish, and encapsulated islet organoids using real‐time PCR, respectively. The expression values are normalized to the GAPDH. Three independent experiments are performed. Data are shown as mean ± SD. **p < 0.01, ***p < 0.001. C) The expressions of INS, NKX6.1, and GCG were identified by immunohistochemical analysis and quantifications for the percentage of INS^+^, NKX6.1^+^, and GCG^+^ cells in islet organoids within capsules. D) Measurements of secreted insulin from human islet organoids that was sequentially exposed to low (2.5 mm) and high glucose (25 mm) during the course of culture in capsules. Data are represented as mean ± SD. Student's *t*‐tests are performed. *p < 0.05.

The insulin response to glucose challenges is closely related to the functionality of islets. The dysfunction of islet tissue may lead to hyperglycemia due to insufficient insulin secretion, ultimately resulting in diabetes. To further assess the functionality of islet organoids, we investigated their ability to perform glucose‐stimulated insulin secretion (GSIS). About 200 islet organoids in hybrid capsules were collected to test GSIS at day 1, 4, and 7 during cultivation, respectively. As shown in Figure 5D, islet organoids maintain insulin secretion for 7 days and show a significantly higher level of insulin secretion in high glucose (25 mm) than that in low glucose (2.5 mm) at 4 and 7 days. These results indicate that the encapsulated islet organoids display insulin secretion functions and sensitive responses to high‐glucose challenges. Also, these results can further validate that small molecules, like glucose (180 Da), are able to enter the hybrid capsules. Taken together, the human islet organoids generated from the hybrid capsules reveal controllable size, proper viability, and islet‐specific functions, which is conducive to reducing the variability and improving the functionalities of organoids. In addition, the capsules for encapsulating islet organoids have the properties of immunoisolation and high stability due to the electrostatic complexation of NaA and CS. As such, the islet organoid encapsulation may provide a potentially translational therapy option for diabetes.

In summary, we introduce a novel strategy for one‐step fabrication of hydrogel capsules in droplet microfluidic system that enables 3D culture, growth, and massive generation of human islet organoids from hiPSCs in a continuous process. The fabricated binary capsules display well‐defined properties with biocompatibility, permeability, stability, and uniformity, which facilitates cell differentiation, self‐organization, and formation of human islet organoids from hiPSCs. The islet organoids produced in the capsules exhibit favorable growth, pancreatic islet‐specific cell types with glucose stimulated insulin secretion functions. The established system can facilitate the reduction of variability of organoids by providing uniform 3D scaffolds in a reproducible manner. This work provides a proof‐of‐concept to engineer human organoids using an integrative strategy by combining materials, droplet microfluidics, and stem cell biology, which offers a robust and scalable platform for organoid research and therapy.

## Experimental Section

See Supporting Information.

## Conflict of Interest

The authors declare no conflict of interest.

## Supporting information

Supporting Information

Supplemental Video 1
