# Supplementary material for: A Droplet Microfluidic System to Fabricate Hybrid Capsules Enabling Stem Cell Organoid Engineering
Source: Adv Sci (Weinh). 2020 Apr 11;7(11):1903739. doi: 10.1002/advs.201903739 (PMC7284190; doi:10.1002/advs.201903739)
Supplement: Supplementary file 1 — Supporting Information [file ADVS-7-1903739-s001.pdf]

Copyright WILEY-VCH Verlag GmbH & Co. KGaA, 69469 Weinheim, Germany, 2020.

ADVANCED SCIENCE
Open Access

Supporting Information

for *Adv. Sci.,* DOI: 10.1002/advs.201903739

A Droplet Microfluidic System to Fabricate Hybrid Capsules Enabling Stem Cell Organoid Engineering

*Haitao Liu, Yaqing Wang, Hui Wang, Mengqian Zhao, Tingting Tao, Xu Zhang, and Jianhua Qin**

Supporting Information

Droplet Microfluidic System to Fabricate Hybrid Capsules Enabling Stem Cell Organoids Engineering

*Haitao Liu‡, Yaqing Wang‡, Hui Wang, Mengqian Zhao, Tingting Tao, Xu Zhang, and Jianhua Qin**

‡ These authors contribute equally

Experimental Section

Materials: Dextran (500 kDa, GE), PEG (20 kDa, Aladdin), Alginate (55 cps, Qingdao Hyzlin Biology Development Co., Ltd), Chitosan (100-200 cps, Aladdin), Glacial acetic acid (Hengxing Chemical Reagent), NaCl (Tianjin Damao Chemical Reagents Factory), Fluorescein isothiocyanate isomer I (FITC) (95%, J&K), Sodium fluorescein (376 Da, Aladdin), BSA-FITC (67 kDa, meilunbio), dextran-FITC (500 kDa, Sigma), polydimethylsiloxane (PDMS, SYLGARD 184) and its curing agent (Dow Corning Corporation), Photoresist (SU-8 3035, MicroChem Corporation). All reagents are used as received.

Microfluidic chip fabrication: The microfluidic chip was fabricated via standard soft lithography and micromolding methods.[1] Briefly, the PDMS at a weight ratio of 10 : 1 was molded from a photoresist templates after being cured at 80 °C for 2 h in an oven to form structured PDMS. Subsequently, the structured PDMS was bonded to a flat PDMS block after punching with a 1.5 mm diameter punch and oxygen plasma treatment. The height and width of the channel are both 200 μm. The pneumatic

single layer membrane (SLM) valve's membrane was designed to 60 μm in thickness and the dispersed flow channel between SLM valve's membrane was 50 μm in width, which could control the generation of droplet templates.

Capsules fabrication: The all-in-water droplet microfluidic system was composed of 17% (w/w) PEG and 15% (w/w) DEX, which were dissolved in deionized water. 1% (w/w) NaA was dissolved in the DEX solution to form core flow. And 1% (w/w) CS was dissolved in the PEG solution with a pH=5 adjusted by acetic acid to form shell flow. A pure 17% (w/w) PEG solution was used as middle flow. NaA, CS, and pure PEG phases were pumped into the corresponding inlets of the SLM integrated chip by using syringe pumps (11 Picro Plus, Harvard). In the droplet generation unit, as SLM valves switched on, dispersed flow channel remained the initial position without deformation, NaA phase flowed continuously to the first junction. When we switched off the valves, valves compressed the dispersed flow channel and a small amount of NaA solution was injected into PEG phase to form droplet templates. Then, droplet templates were delivered into CS phase by PEG phase at the second junction, wherein NaA and CS diffused to interact at the interface of droplet. Finally, NaA-CS hybrid capsules were fabricated in the reaction unit.

Scanning electron microscope (SEM): Hydrous hybrid capsules were freeze-dried in a lyophilizer (SCIENTZ-10ND, China) over night. Then they were coated with gold for 220 s with a sputter coater (SBC-12, KYKY) after being fixed on an aluminum stub with conductive adhesive tape to improve their electrical conductivity. The size and

surface morphology of the capsules was characterized using a SEM (Hitachi TM3000, Japan) at 15 kV.

FITC labeled chitosan: To label the CS layer in the capsules, FITC was used to tag CS component. The FITC-labeled CS (FITC-CS) was synthesized based on the reaction between the -N=C=S group of FITC and the -NH2 group of CS.[2] Briefly, 0.5 g chitosan was dissolved in 50 mL acetic acid (0.1 M) before adding 50 mL methanol and 25 mL methanol containing FITC (2 mg mL^{-1}). After 3 h of reaction in the dark at room temperature, FITC-CS was precipitated by adding 0.2 M NaOH until the pH raised to 9–10. Unreacted FITC was washed away by a mixture of methanol and water in a 7 : 3 volume ratio separated by centrifugation (17800 g, 10 min) until no fluorescence was detected in the supernatant. FITC-CS was then dialyzed using a dialysis bag (cut-off MW = 7000) in 3 L of distilled water for 3 days in the dark. The distilled water was replaced with fresh distilled water daily. The dialyzed FITC-CS was freeze-dried in a lyophilizer for 3 days for the following experiments. The fluorescence images of FITC labeled capsules were acquired with a fluorescence microscopy (Olympus IX71, Japan).

Diffusion experiments: The diffusion process through the shell of hybrid capsules was detected using 5 mM fluorescein sodium (376 Da), 2.5 mg mL^{-1} BSA-FITC (67 kDa), and 5 mg mL^{-1} dextran-FITC (500 kDa), representing small molecules, proteins, and macromolecule, respectively. The newly fabricated capsules were immersed in solutions of these fluorescent molecules that might diffuse across the shell to enter the capsules, followed by investigation of an area that was 100 μm larger of diameter than

the capsule under a confocal microscope, immediately (Olympus IX 81, Japan). The fluorescence images of diffusion process were taken every 5 min in the first 30 minutes, then taken every 10 min in the next investigation. The fluorescein sodium experiment lasted for 80 min, while the BAS-FITC and dextran-FITC experiment continued for 120 min. The absolute fluorescence intensity was quantified using Image-Pro Plus 6.0 software. The fluorescence intensity in the capsules was normalized with the fluorescence intensity of the area outside the capsules.[3, 4] To calculate the permeability value, we set a background area (a round that is 100 μm larger of diameter than the capsule). Then, $P = \frac{D\times(F_E-F_0)}{F_t\times T_E}$ (P being permeability value, D (μm) being diameter of capsules, F_E being the fluorescence intensity in capsules at equilibrium state, F_0 being the fluorescence intensity in capsules at 0 min, F_t being the total fluorescence intensity in the selected area at 0 min, T_E (s) being the time for diffusion equilibrium).

Cell culture: Rat islet cell line (β-TC6) was purchased from the Cell Bank of the Chinese Academy of Sciences (Kunming, China). The β-TC6 cells were cultured in Dulbecco's Modified Eagle Medium (DMEM, low glucose, Gibco) supplemented with 1% streptomycin and penicillin (Biyotime), 10% fetal bovine serum (FBS, Gibco). They were cultured in a humidified atmosphere composed of 5% CO_2 at 37 ^oC and passaged until achieved 80%-90% confluence. To demonstrate the stability of hydrogel capsules for 3D cells culture, the newly fabricated FITC-labeled capsules were incubated under aforementioned cell culture conditions for 25 days before cells encapsulation experiment.

Human iPSCs culture and differentiation: The hiPSCs used in this study were kindly provided by Professor Ning Sun (Fudan University, Shanghai, China).[5-7] Undifferentiated hiPSCs were cultured in Matrigel-coated six-well plates (1 : 50 dilution, BD) with mTeSR1 medium (StemCell) in a humidified incubator with 5% CO_2, wherein the fresh medium was changed daily. To generate pancreatic endocrine cells, iPSCs were induced by sequential addition of various growth factors when reached about 60% to 70% confluence in petri dish. The differentiation protocol was modified according to a previous report.[8] For definitive endoderm (DE) induction, iPSCs were incubated in medium containing basal DMEM/F12 (Invitrogen), 1% KSR (Invitrogen), 1% B27 supplement (Invitrogen), 100 ng mL^{-1} activin-A (PeproTech), 1% GlutaMAX (Invitrogen) and 1% penicillin-streptomycin (Sigma) for 5 days. For pancreatic endoderm (PE) differentiation, cells were cultured in medium containing DMEM (Invitrogen), 0.5% B27 supplement, 2 μM dorsomorphin (Selleck), 2 μM retinoic acid (RA, Sigma), 10 μM SB431542 (Selleck), 5 ng mL^{-1} basic fibroblast growth factor (bFGF; R&D systems), and 250 nM SANT-1 (Selleck) for 6 days. Then, to facilitate endocrine progenitor (EP) cell differentiation, the PE cells were cultured in DMEM containing 0.5% B27 supplement, 2 μM dorsomorphin, 10 μM SB431542, 50 μg mL^{-1} ascorbic acid (Sigma), and 10 μM DAPT (Abcam Biochemicals). After day 15, the medium was replaced with CMRL 1066 (Invitrogen) containing 25 mM glucose (Sigma), 0.5% B27 supplement, 10 mM nicotinamide (Sigma), 10 μM SB431542, 50 μg mL^{-1} ascorbic acid and 2 μM dorsomorphin for a long-term culture of islet cells. This article does not contain any studies with human participants or

animals performed by any of the authors. All experiments were performed in compliance with the relevant laws and institutional guidelines. The institutional committee have approved the experiments.

Cell encapsulation: β-TC6 cells and pancreatic endocrine cells at day 20 of differentiation from iPSCs were dissociated by 0.25% trypsin-EDTA (Gibco), then resuspended in core flow solution with a density of 4×10^6 cells mL^{-1} (β-TC6) and $3\sim5\times10^7$ cells mL^{-1} (pancreatic endocrine cells) for encapsulation experiments, respectively. Here, the core flow solution was prepared by mixing cell suspensions with a concentrated NaA solution (22.5% (w/w) DEX and 1.5% (w/w) NaA dissolved in physiological saline) in a 1 : 2 ratio. The final concentration of solution contained 15% (w/w) DEX and 1% (w/w) NaA 17% (w/w) PEG and 17% (w/w) PEG with 1% (w/w) CS were also dissolved in physiological saline as the middle and shell flow, respectively. All these solutions were filtered with a 0.22 μm syringe filter (Millex Syringe Filter) before being injected into the device using syringe pumps with 25mm long Teflon tubes (ID 0.75 mm, Beijing Jieanjie Technology Development Co., Ltd.). The generated cell-laden capsules were collected in physiological saline sterilized with autoclave (MLS-3751L, Panasonic, Japan), followed by natural sedimentation in centrifuge tubes for 5 min. The cells in capsules were finally transferred to cell culture medium in a 24-well culture plate and cultured in a humidified atmosphere of 5% CO_2 at 37 ^{o}C with the fresh medium being changed daily.

Cell viability: Cell viability of encapsulated cells was evaluated by using live/dead assay. The cell-laden capsules were incubated in cell culture medium with ethidium

homodimer-1 (red, dead, 1 : 500) and calcein-AM (green, live, 1 : 1000) (LIVE/DEAD viability/cytotoxicity assay kit, Gibco) at 37 °C for 25 min. Then, physiological saline was introduced for a final rinse before imaging under a fluorescence microscopy (Leica DMI3000 B, Germany). The percentage viability of encapsulated cells was determined by measuring the area of green fluorescence (live cells), which was divided by the total area of green + red fluorescence (dead cells) at a given focal plane.

CCK-8 assays: A cell counting kit-8 (CCK-8, Dojindo, CK04) assay was used to assess cellular growth vitality. Here, the encapsulated β-TC6 cells with an initial density of 2×10^{4} cells mL^{-1} were cultured in a 96-well culture plate. Then, the culture medium was replaced by 10% CCK-8 medium (100μL each) at days 2, 4, 6, and 8 before incubation for 2 h at 37 °C. Finally, the 96-well plate was put into a microplate reader (Tecan Infinite M Nano, Switzerland) to measure the absorbance at 450 nm.

Fluorescence immunohistochemistry: β-TC6 cells in capsules were soaked in 4% paraformaldehyde (PFA) (Tianjin Damao Chemical Reagents Factory) for 15 min to be immobilized after cultivation of 8 days. Then they were permeabilized with 0.2% Triton X-100 (Amresco) for 10 min and blocked in a blocking solution containing 10% goat serum for 2 h at room temperature, successively. Next, the cells were incubated at 4 °C overnight with primary rabbit anti-insulin (diluted 1 : 100) antibodies (Cell Signating Technology), which were then exposed to secondary antibodies (diluted 1 : 500) (Alexafluor ®488) for 1 hour at room temperature. The cell nuclei were stained with DAPI (1 : 4000, CST 4083) for 15 min at room

temperature. For the encapsulated islet organoids, they were collected after encapsulation of 7 days. Then, they were fixed, permeabilized, blocked, and incubated with primary antibodies as described above. Here, the primary antibodies were as follows: insulin (Guinea pig, 1 : 100, ab7842), GCG (rabbit, 1 : 400, Bioss bs-3796R), PPY (rabbit, 1 : 100, GTX128056), and NKX6.1 (rabbit, 1:250, CST D804R). The organoids were then incubated with Alexa Fluor 488- or 594-conjugated secondary antibodies (1 : 500) at RT for 1 h. DAPI (1 : 4000) was used to stain the cell nuclei. For the undifferentiated iPSCs, they were collected when reached about 60% to 70% confluence in petri dish. Then, they were fixed, permeabilized, blocked, and incubated with primary antibodies as described above. The primary antibodies were as follows: insulin (Guinea pig, 1 : 100, ab7842), GCG (rabbit, 1 : 400, Bioss bs-3796R), PPY (rabbit, 1 : 100, GTX128056), SST (rat, 1 : 100, ab30788), OCT4A (rebbit, 1 : 500, Cell Signaling 2890S), and SOX2 (rabbit, 1 : 500, Cell Signaling 3579). The cells then incubated with Alexa Fluor 488- or 594-conjugated secondary antibodies (1 : 500) at RT for 1 h. DAPI (1 : 4000) was used to stain the cell nuclei. All the images were acquired with a confocal microscope. The percentages of INS^{+}, $NKX6.1^{+}$ and GCG^{+} cells in islet organoids within capsules were obtained by calculating the ratio of fluorescent area of INS, NKX6.1 and GCG to DAPI at a given focal plane.

Real-time quantitative PCR: The whole mRNA was extracted from islet organoids in capsules using a Trizol reagent (TAKARA) to detect the associated genes expression level. The final concentration of mRNA was adjusted to 250 ng mL^{-1}. The cDNA was then synthesized by reverse transcription polymerase chain reaction (RT-PCR)

(TAKARA). Subsequently, real-time quantitative PCR was performed using SYBR Green kit (TAKARA) under the following reaction conditions (40 cycles): denaturation at 95 °C for 30 s, annealing at 58 °C for 30 s, and extension at 72 °C for 30 s. The primer pairs were as follows: INS forward: 5′-TGT ACC AGC ATC TGC TCC CTC TA-3′, reverse: 5′-TGC TGG TTC AAG GGC TTT ATT CCA-3′; NKX6.1 forward: 5′-ATT CGT TGG GGA TGA CAG AG-3′, reverse: 5′-TGG GAT CCA GAG GCT TAT TG-3′; GCG forward: 5′-AGG CAG ACC CAC TCA GTG A-3′, reverse: 5′-AAC AAT GGC GAC CTC TTC TG-3′; PPY forward: 5′- ACC TGC GTG GCT CTG TTA CT-3′, reverse: 5′-TAC CTA GGC CTG GTC AGC AT-3′. Quantification was performed using GAPDH as the reference gene.

Glucose-stimulated insulin secretion assay: To assess the physiological function of encapsulated cells, the glucose-stimulated insulin secretion was measured at days 1, 4, and 7. Approximately 200 organoid-laden capsules were collected into a centrifuge tube, followed by wash with Krebs-Ringer bicarbonate HEPES (KRBH) buffer for 3 times. Initially, these organoids were cultured in 2.5 mM glucose KRBH for 2 h to remove residual insulin. Then, organoids were sequentially incubated in 2.5 mM and 25 mM glucose KRBH (500 μL each) for 30 min with wash using fresh 2.5 mM glucose KRBH for 3 times between each procedure. Finally, the supernatants were collected and frozen at -80 ^{o}C until measuring the concentration of insulin with sandwich enzymatic ELISA kits (Abebio). Insulin concentration was obtained by measuring the absorbance at 450 nm and expressed as mIU L^{-1}.

Size measurement and distribution statistics: The images of free capsules were acquired in the physiological saline bath with an inverted microscope (Olympus IX-71, Japan). The images of cell-laden capsules were acquired in the cell culture medium with an inverted microscope (Leica DMi8, Germany). The implementation of size measurement and distribution statistics of capsules and cellular spheroids are using Image J software (http://rsb.info.nih.gov/ij/).

Statistic: In this work, all experiments were performed at least in triplicate. All the data were presented as mean ± standard deviation (SD), unless stated differently. Statistical significance was declared when *$p < 0.05$, **$p < 0.01$ and ***$p < 0.001$ as determined by one-way ANOVA and student's t-test.

References

[1] H. T. Liu, H. Wang, W. B. Wei, H. Liu, L. Jiang, J. H. Qin. *Small* **2018**, *14*.

[2] M. Huang, E. Khor, L. Y. Lim. *Pharmaceutical Research* **2004**, *21*, 344.

[3] B. Prabhakarpandian, M. C. Shen, J. B. Nichols, I. R. Mills, M. Sidoryk-Wegrzynowicz, M. Aschner, K. Pant. *Lab Chip* **2013**, *13*, 1093.

[4] F. C. Yin, Y. J. Zhu, M. Zhang, H. Yu, W. W. Chen, J. H. Qin. *Toxicol. In Vitro* **2019**, *54*, 105.

[5] L. Wang, C. Xu, Y. J. Zhu, Y. Yu, N. Sun, X. Q. Zhang, K. Feng, J. H. Qin. *Lab Chip* **2015**, *15*, 4283.

[6] Q. J. Wang, H. Yang, A. B. Bai, W. Jiang, X. Y. Li, X. H. Wang, Y. S. Mao, C. Lu, R. Z. Qian, F. Guo, T. L. Ding, H. Y. Chen, S. F. Chen, J. Y. Zhang, C. Liu, N. Sun.

Biomaterials **2016**, *105*, 52.

[7] N. Sun, N. J. Panetta, D. M. Gupta, K. D. Wilson, A. Lee, F. Jia, S. Hu, A. M. Cherry, R. C. Robbins, M. T. Longaker, J. C. Wu. *Proc. Natl. Acad. Sci. U. S. A.* **2009**, *106*, 15720.

[8] T. T. Tao, Y. Q. Wang, W. W. Chen, Z. Y. Li, W. T. Su, Y. Q. Guo, P. W. Deng, J. H. Qin. *Lab Chip* **2019**, *19*, 948.

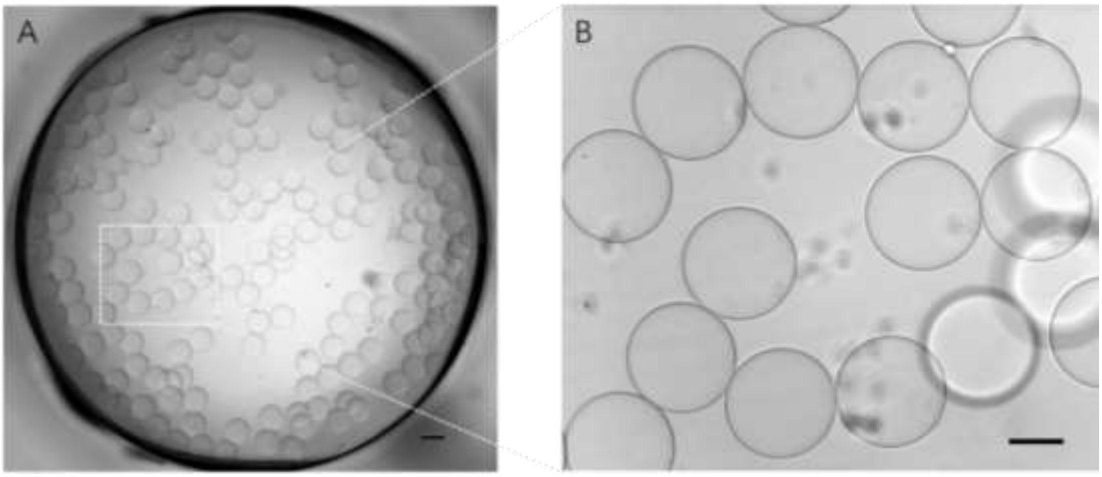

Figure S1. Large-scale production of capsules in the all-in-water droplet microfluidic system. A. massive capsules collected in 384-well plate. Scale bar: 200μm. B. Enlarge image of A. Scale bar: 100μm. These capsules are fabricated under the same conditions (rates of core, middle and shell flow: 0.2, 2, and 4 μL min^{-1}; valve frequency: 0.4 s; concentrations of NaA /CS: 1% (w/w)).

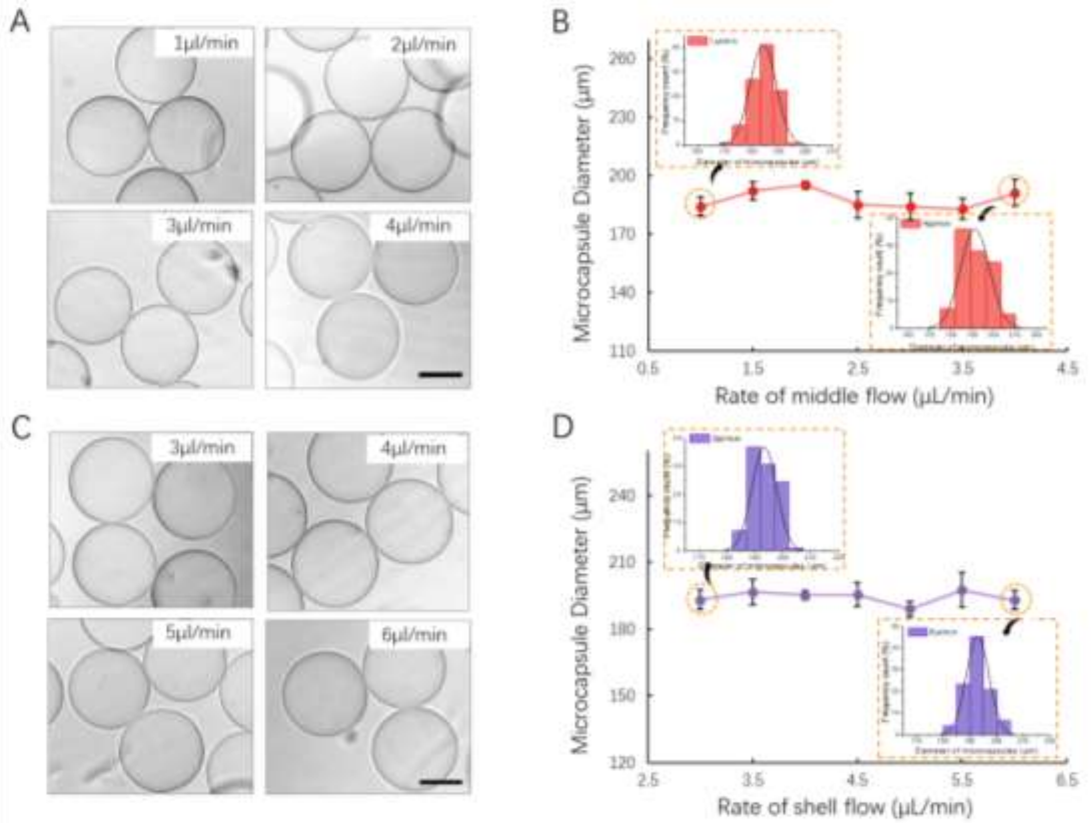

Figure S2. Effects of middle and shell flow rates on diameter and polydispersity of the generated capsules. The images of capsules generated under different flow rates of (A) middle flow (1-4 μL min^{-1}) and (C) shell flow (3-6 μL min^{-1}), respectively. The diameter as a function of flow rate of (B) meddle flow and (D) shell flow and size distribution of capsules generated under boundary flow rates (1 and 4 μL min^{-1} for middle flow; 3 and 6 μL min^{-1} for shell flow). Quantitative analysis of the diameter of capsules were performed on at least 50 capsules. Scale bars: 100μm.

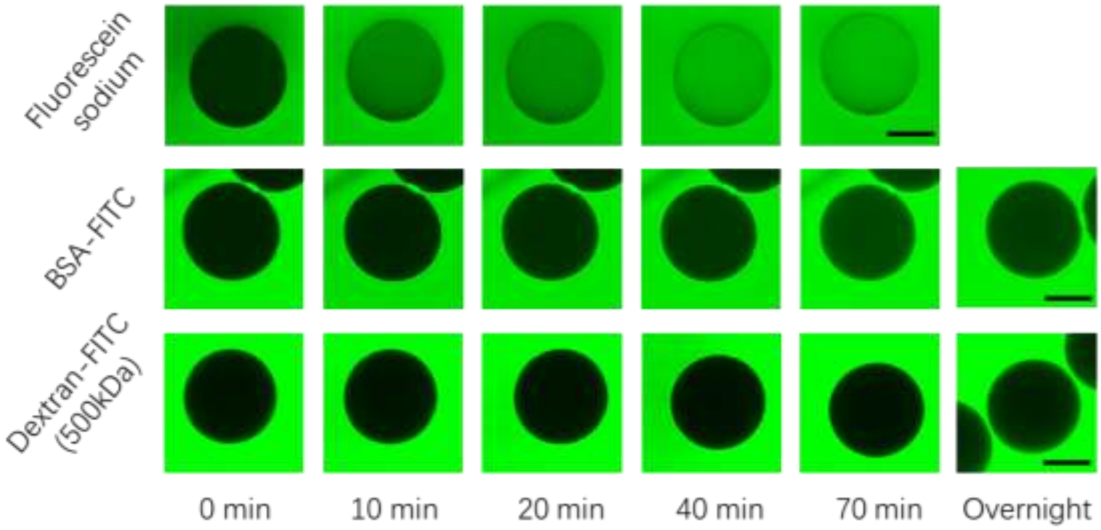

Figure S3. Fluorescence images of fluorescein sodium, BSA-FITC, and dextran-FITC (500kDa) diffuse in hybrid capsules. Scale bars: 100μm. These capsules are fabricated under the same conditions (rates of core, middle and shell flow: 0.15, 2, and 4 μL min^{-1}; valve frequency: 0.4 s; concentrations of NaA /CS: 1% (w/w)).

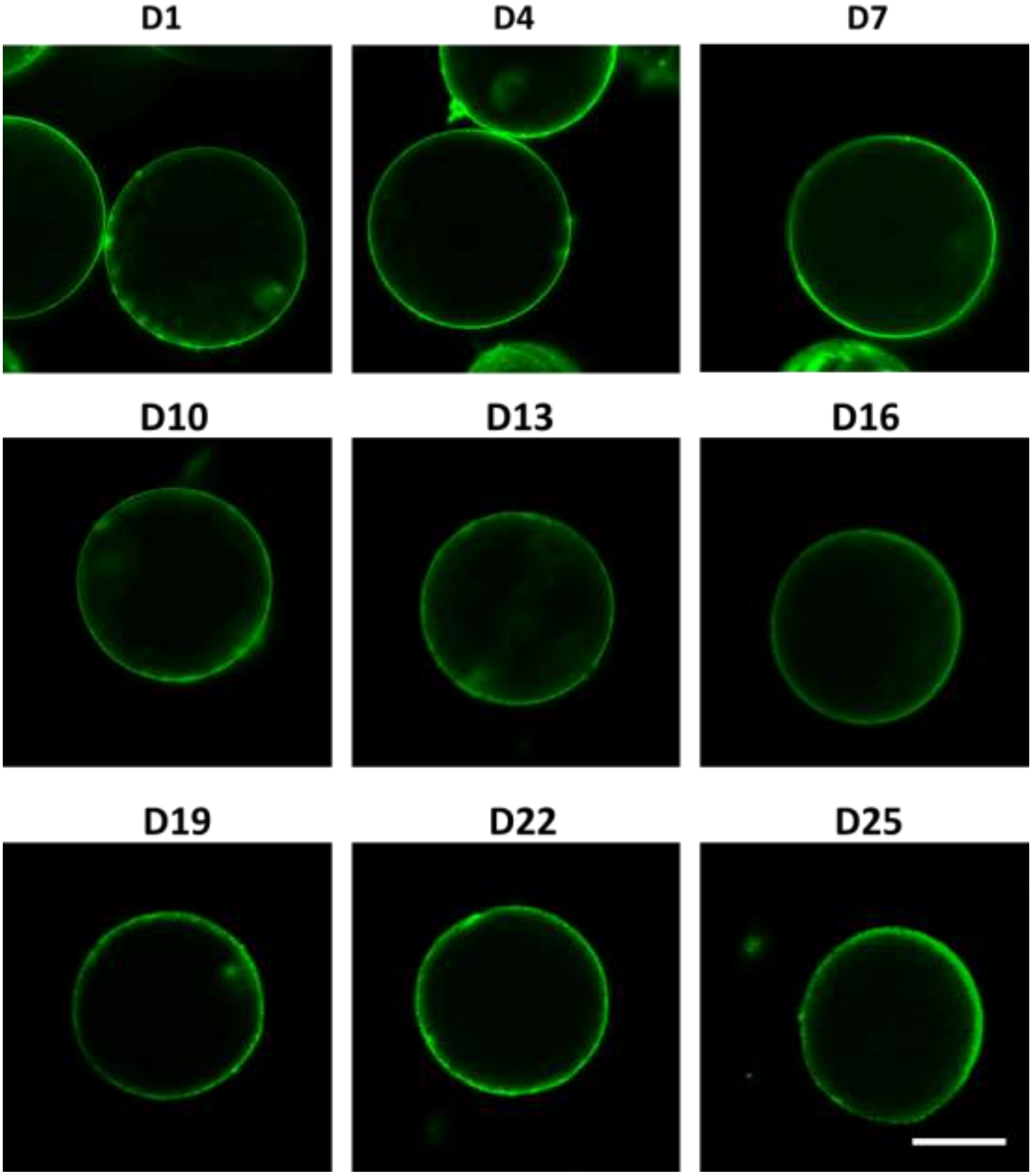

Figure S4. The integrity of hybrid hydrogel capsules under cell culture conditions. Such capsules are fabricated with FITC-chitosan, which are immersed in DMEM medium supplemented with 1% streptomycin and penicillin, 10% fetal bovine serum in a humidified atmosphere composed of 5% CO_2 at 37 ^oC for 25 days. Scale bars: 100μm. These capsules are fabricated under the same conditions (rates of core, middle and shell flow: 0.15, 2, and 4 μL min^{-1}; valve frequency: 0.4 s; concentrations of NaA /FTIC-CS: 1% (w/w)).

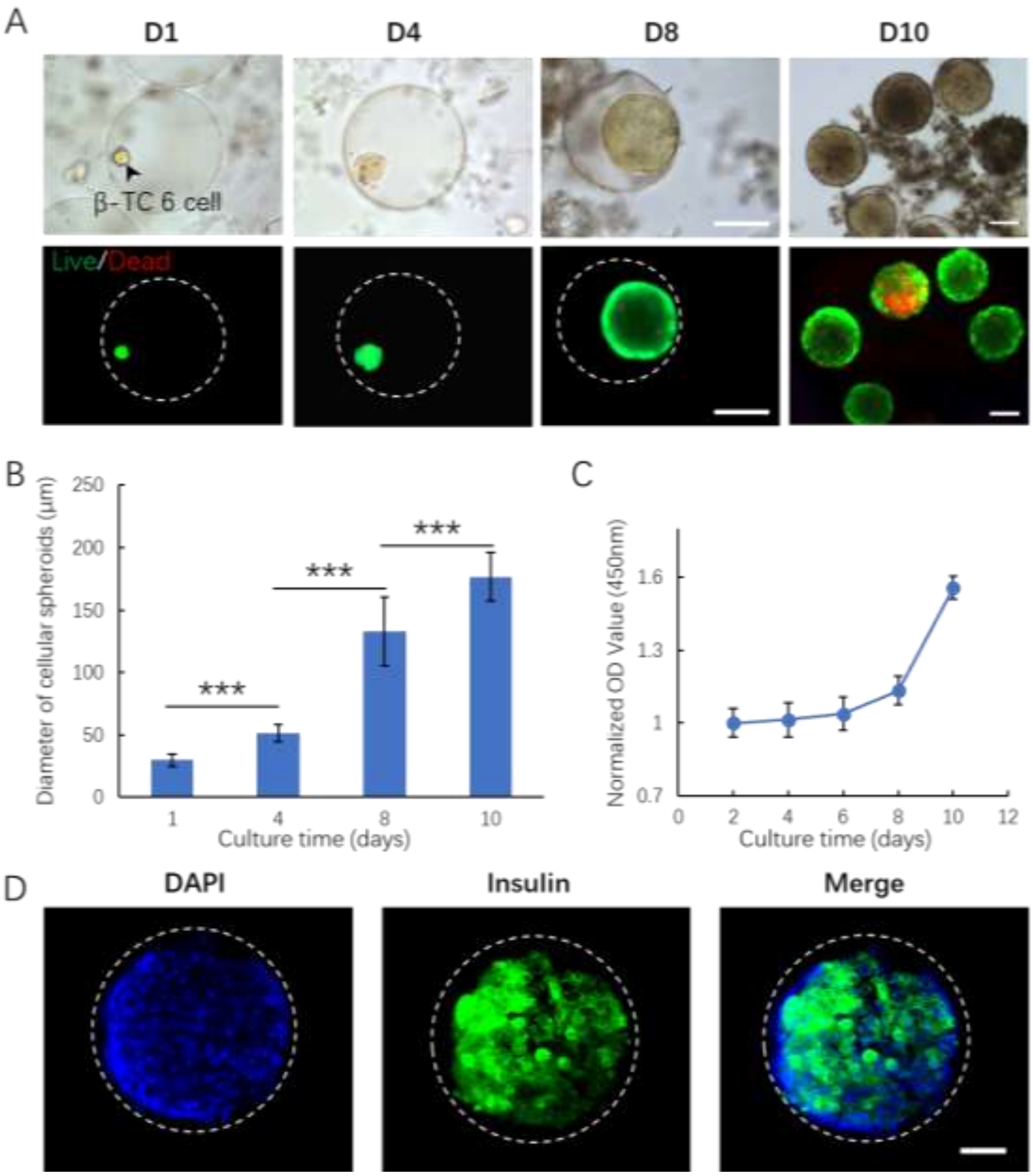

Figure S5. Encapsulation and 3D culture of β-TC6 cells in hybrid capsules. (A) Bright-field images and cell viability of spheroids in hybrid capsules after encapsulation of 1, 4, 8 and 10 days. The green and red fluorescence represent live and dead cells, respectively. Scale bars: 100μm. (B) Size distribution was assessed by the diameter of spheroids in capsules on days 1, 4, 8 and 10. Quantitative analysis of the diameter of cellular spheroids were performed on at least 15 spheroids. Data are shown as mean ± SD. Student's t-tests were performed, ***p < 0.001. (C) The proliferation ability of encapsulated β-TC6 cells was examined by CCK8 assays. (D) Immunofluorescence staining for insulin in β-TC6 cell spheroids in capsules at day 10. Blue fluorescence represents nucleus and green fluorescence represents secreted insulin. Scale bar: 50μm.

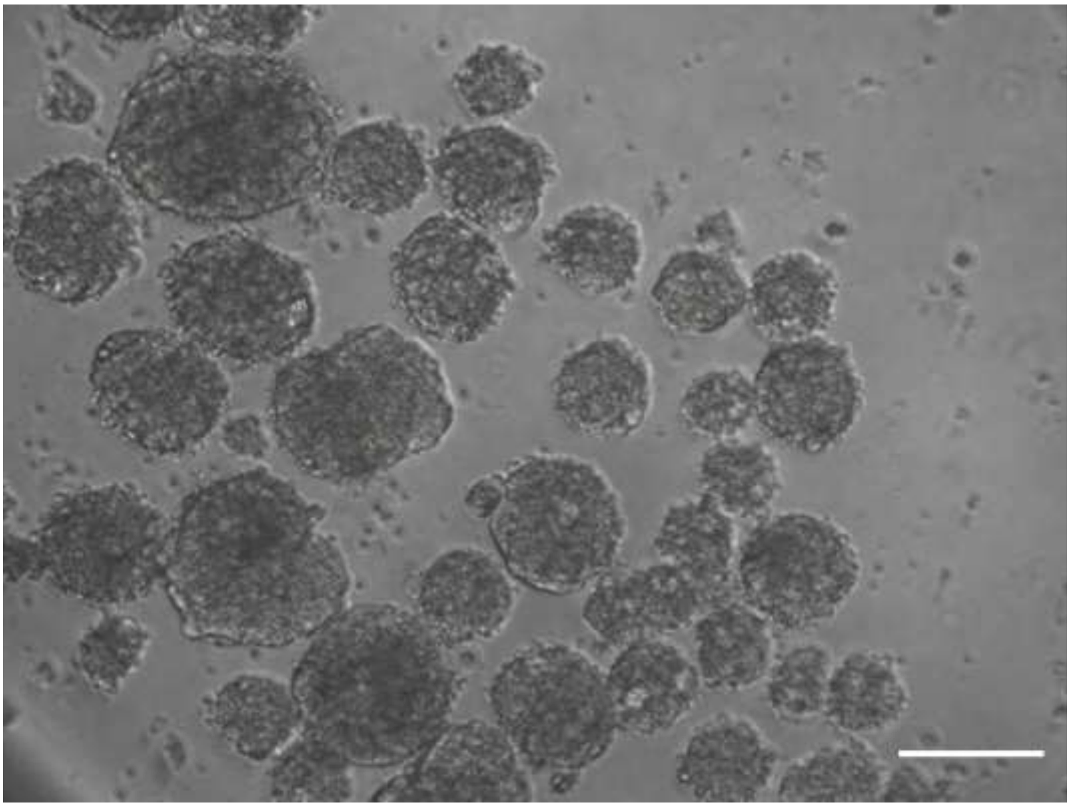

Figure S6. Islet organoids were formed by randomly organization of islet cells at a density of 5×10^4 cells mL^{-1} in low-adhesion Petri dish. These organoids exhibited marked variability of size. Scale bar: 50 μm.

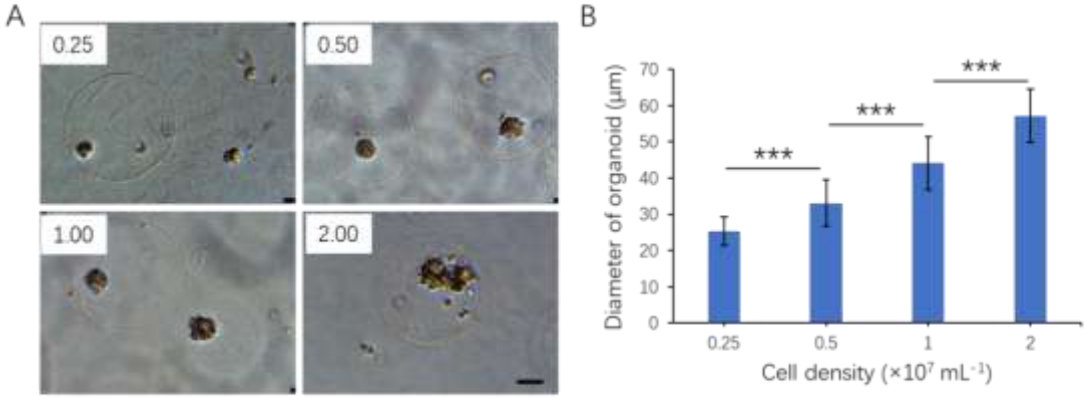

Figure S7. Islet organoids with varied size were generated with different loaded islet cell densities in the core flow. (A) Bright-field images of islet organoids generated with different islet cell densities (0.25, 0.50, 1.00 and 2.00×10^7 cells mL^{-1}). The size of organoids varied as the cell density changes. Scale bar: 50 μm (B) Quantitative analysis of islet organoids with different size as cell density changes (0.25, 0.50, 1.00 and 2.00×10^7 cells mL^{-1}). Student's t-tests were performed, ***$p < 0.001$.

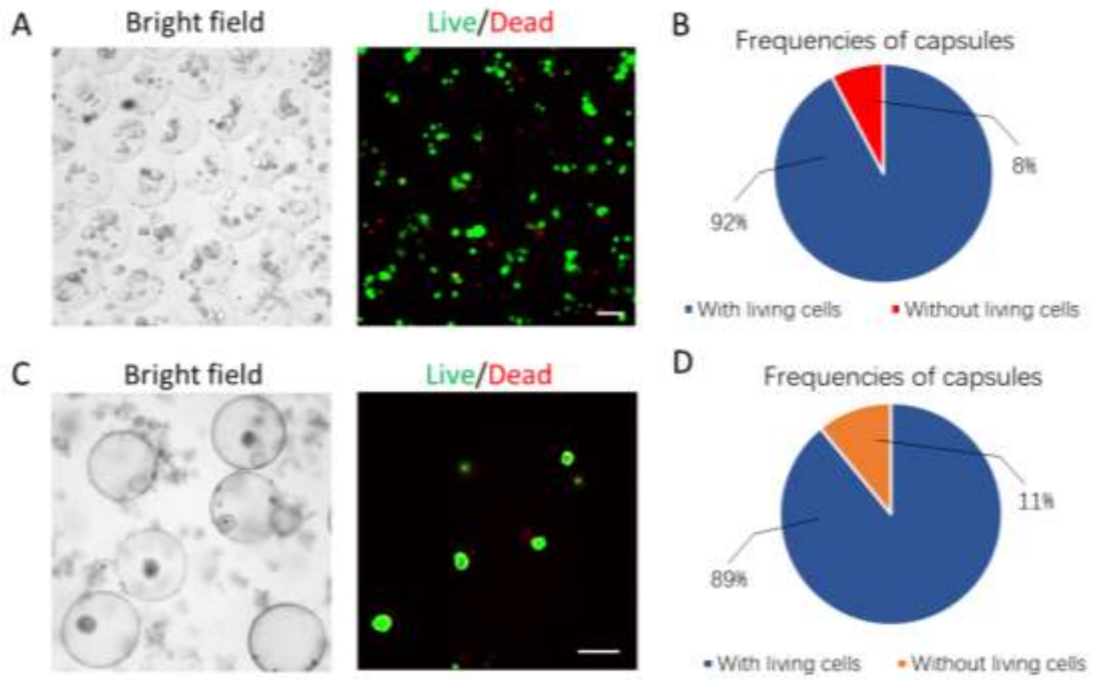

Figure S8. The frequencies of capsules with/without living cells at days 0 and 7. (A) Representative bright-field and Live/Dead staining images of islet cell spheroids after encapsulation in capsules at day 0. The green and red fluorescence represent live and dead cells, respectively. Scale bars: 100µm. (B) The frequencies of capsules with/without living cells at day 0. (C) Representative bright-field and Live/Dead staining images of islet cell spheroids after encapsulation in capsules at day 7. The green and red fluorescence represent live and dead cells, respectively. Scale bars: 100µm. (D) The frequencies of capsules with/without living cells at day 7.

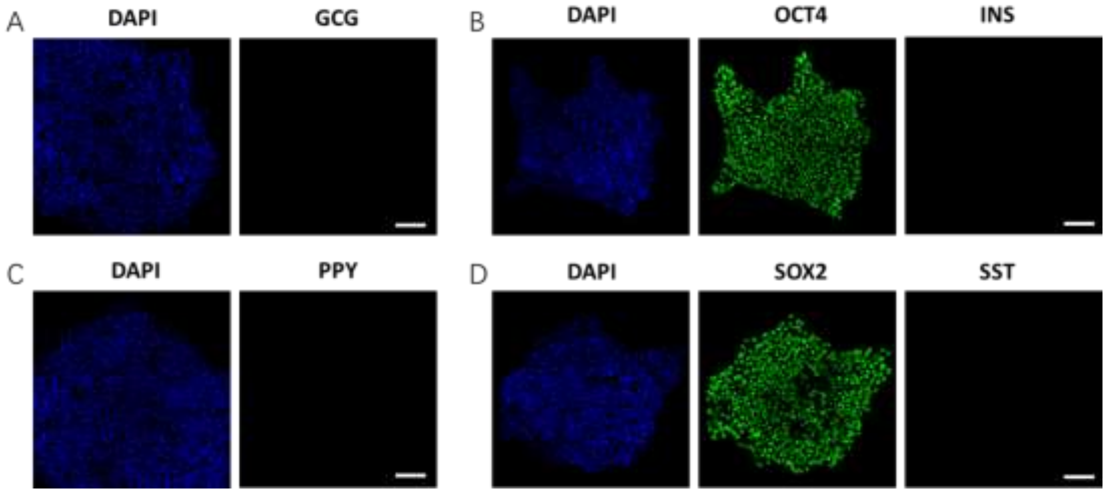

Figure S9. Immunohistochemical staining of pancreatic endocrine hormone markers and stem cell markers in undifferentiated iPSCs. (A) Immunohistochemical staining of pancreatic endocrine hormone marker GCG in undifferentiated iPSCs. (B) Immunohistochemical staining of pancreatic endocrine hormone marker INS and stem cell marker OCT4 in undifferentiated iPSCs. (C) Immunohistochemical staining of pancreatic endocrine hormone marker PPY in undifferentiated iPSCs. (D) Immunohistochemical staining of pancreatic endocrine hormone marker SST and stem cell marker SOX2 in undifferentiated iPSCs. Scale bars: 50 μm.
